# Dr. John Charles Maloney

**Published:** 1915-11

**Authors:** 


**Dr. John Charles Maloney,** Yale 1910, died at his home in Dundee, Aug. 1, aged 29.

